# The Effect of Polyethylene Terephthalate Nanoplastics on Amyloid-β Peptide Fibrillation

**DOI:** 10.3390/molecules30071432

**Published:** 2025-03-24

**Authors:** Narmin Bashirova, Franziska Schölzel, Dominik Hornig, Holger A. Scheidt, Martin Krueger, Georgeta Salvan, Daniel Huster, Joerg Matysik, A. Alia

**Affiliations:** 1 Institute of Medical Physics and Biophysics, Leipzig University, D-04107 Leipzig, Germanydaniel.huster@medizin.uni-leipzig.de (D.H.); 2 Institute of Analytical Chemistry, Leipzig University, D-04103 Leipzig, Germany; joerg.matysik@uni-leipzig.de; 3 Institute of Physics, Chemnitz University of Technology, D-09126 Chemnitz, Germanysalvan@physik.tu-chemnitz.de (G.S.); 4 Center for Materials, Architectures and Integration of Nanomembranes, Chemnitz University of Technology, D-09126 Chemnitz, Germany; 5 Institute of Chemistry, Chemnitz University of Technology, D-09107 Chemnitz, Germany; 6 Institute of Anatomy, Leipzig University, D-04107 Leipzig, Germany; 7 Leiden Institute of Chemistry, Leiden University, 2333 CC Leiden, The Netherlands

**Keywords:** polyethylene terephthalate, nanoplastics, amyloid β, fibrillation

## Abstract

Exposure of organisms to nanoplastics (NPs) is inevitable given their global abundance and environmental persistence. Polyethylene terephthalate (PET) is a common plastic used in a wide range of products, including clothing and food and beverage packaging. Recent studies suggest that NPs can cross the blood-brain barrier and cause potential neurotoxicity. It is widely known that aggregation of amyloid beta (Aβ) peptides in the brain is a pathological hallmark of Alzheimer’s disease (AD). While the impact of nanoplastics such as polystyrene (PS) on amyloid aggregation has been studied, the effects of PET NPs remain unexplored. In this study, we examined the effect of PET NPs of different sizes (PET_50nm_ and PET_140nm_) and concentrations (0, 10, 50, and 100 ppm) on the fibrillation of Aβ_1-40_. Our results showed that the presence of PET_50nm_ as well as PET_140nm_ decreased the lag phase of the fibrillation processes in a dose- and size-dependent manner from 6.7 ± 0.08 h for Aβ in the absence of PET (Aβ_control_) to 3.1 ± 0.03 h for PET_50nm_ and 3.8 ± 0.06 h for PET_140nm_. CD spectroscopy showed that PET_50nm_ significantly impacts the structural composition of Aβ aggregates. A significant rise in antiparallel β-sheet content and β-turn structure and a substantial reduction in other structures were observed in the presence of 100 ppm PET_50nm_. These changes indicate that higher concentrations (100 ppm) of PET_50nm_ promote more rigid and uniform peptide aggregates. Although PET_50nm_ NPs influence the kinetics of aggregation and secondary structure, the overall morphology of the resulting fibrils remains largely unaltered, as seen using transmission electron microscopy. Also, the local cross-β structure of the fibrils was not affected by the presence of PET_50nm_ NPs during fibrillation, as confirmed using ^13^C solid-state NMR spectroscopy. Overall, these findings show that PET NPs accelerate amyloid fibril formation and alter the secondary structure of Aβ fibrils. These results also indicate that the accumulation of PET-NPs in the brain may facilitate the progression of various neurodegenerative diseases, including Alzheimer’s disease.

## 1. Introduction

Single-use plastic products for food and beverage packaging enjoy high popularity and the global production of plastics keeps increasing along with its detrimental impact on the environment. As of 2022, the world produces nearly 400 million tons of plastic annually (OECD report [1]). The most abundant plastic polymers are polyethylene (PE), polypropylene (PP), polyvinyl chloride (PVC), polyethylene terephthalate (PET), and polystyrene (PS). Of the total production of plastics, about 70% ends up in landfills or is incinerated, only around 10% is recycled, and the remaining 20% ends up in the environment. Global plastic waste is expected to almost triple by 2060, reaching over 1000 million tons per year if current trends continue (OECD report [1]).

All this plastic material eventually breaks down into smaller particles, which are further dispersed into terrestrial and marine environments [2,3,4]. The degradation of plastics can occur through various mechanisms, including thermal degradation, photodegradation, chemical degradation, and biodegradation [5,6,7,8]. During the degradation process, plastics release smaller fragments, called micro- or nanoplastics (MNPs) [9]. Nanoplastics (NPs), defined as plastic particles smaller than 1 micrometer, have emerged as environmental and health concerns due to their ability to interact with biological systems in various ways. Given their size, these particles can be internalized by cells and may alter cellular structures such as the cell membrane at the molecular level [10].

Nanoplastics can enter the human body through ingestion of contaminated food and water or inhalation of airborne particles [11,12]. Research indicates that NPs can cross biological barriers such as the intestinal lining and lung epithelium, allowing them to enter the bloodstream and distribute to various organs [13,14]. In the bloodstream, NPs are transported to different tissues and may potentially cross the blood-brain barrier (BBB) [15]. Once NPs cross the BBB, they can accumulate in brain tissue, which may lead to neuroinflammation, oxidative stress, and neurotoxicity [16,17,18]. These effects might impair brain function and contribute to neurological disorders. Although the full extent of their impact on human health is still being researched, the presence of NPs in the brain raises significant concerns about long-term health risks [18,19,20]. The widespread presence of NPs has been linked to several conditions such as obesity, diabetes, cognitive impairment, and neurodegenerative diseases including Alzheimer’s disease (AD) and Parkinson’s disease [21].

PET is a type of plastic widely used in various industries, primarily in food and beverage packaging, as well as in textiles [22,23]. PET is valued for its strength, flexibility, durability, and resistance to chemicals and moisture, making it an ideal material for a wide range of consumer products [24]. Recent studies have demonstrated that PET NPs exhibit neurotoxic effects in zebrafish embryos [24]. Exposure to PET NPs at a concentration of 100 ppm resulted in increased hypoactivity [24]. Additionally, Nile red staining revealed the accumulation of these particles in the zebrafish brain. Metabolomics analysis showed disruptions in energy metabolism (glucose, acetate, ATP, NADH, lactate) and neurotransmitter pathways (γ-aminobutyric acid (GABA), glutamate, glutamine) due to PET NP exposure [24]. Given the established impact of PET NPs on zebrafish embryos, it becomes crucial to explore their potential effects on human neurological health, particularly in the context of neurodegenerative diseases. AD is a neurodegenerative disease that manifests as a progressive decline in cognitive functioning, memory loss, and behavioral changes [25]. It is a common cause of dementia among the elderly, affecting 30 million individuals worldwide [25]. Although the abnormal aggregation of amyloid peptides has been identified as a primary pathological mechanism in AD, there is growing concern that the increase in these neurological disorders may also be driven by exposure to ubiquitous but poorly understood environmental contaminants. In a recent study, Gou et al. [20] studied the impact of polystyrene (PS) nanoparticles on Alzheimer ’s disease and showed that PS NPs accelerate amyloid β peptide (Aβ) aggregation and promote neurotoxicity, thus offering insights into the role of nanoplastic in AD.

Although the effect of other nanoplastics, such as polystyrene (PS), on amyloid aggregation has previously been explored [20], studies on the effect of PET NPs on amyloid aggregation are lacking. Understanding of whether PET NPs may influence Aβ aggregation is important for assessing their potential role in neurodegenerative diseases. Here, we provide a proof-of-concept study to examine the impact of PET NPs on Aβ aggregation kinetics, aggregate morphology, and secondary structural changes. By investigating these interactions, we aim to elucidate the effect of PET NPs on human health and their potential contribution to AD.

## 2. Results and Discussion

In this work, we investigated the effect of PET NPs on Aβ aggregation. PET nanoparticles (PET NPs) with sizes of 50 nm (PET_50nm_) and 140 nm (PET_140nm_) were prepared as described in Section 3. The suspension of PET NPs of both sizes remained stable for several weeks without any settling even at a concentration as high as 1 mg/mL.

### 2.1. Characterization of PET NPs

**Figure 1:**
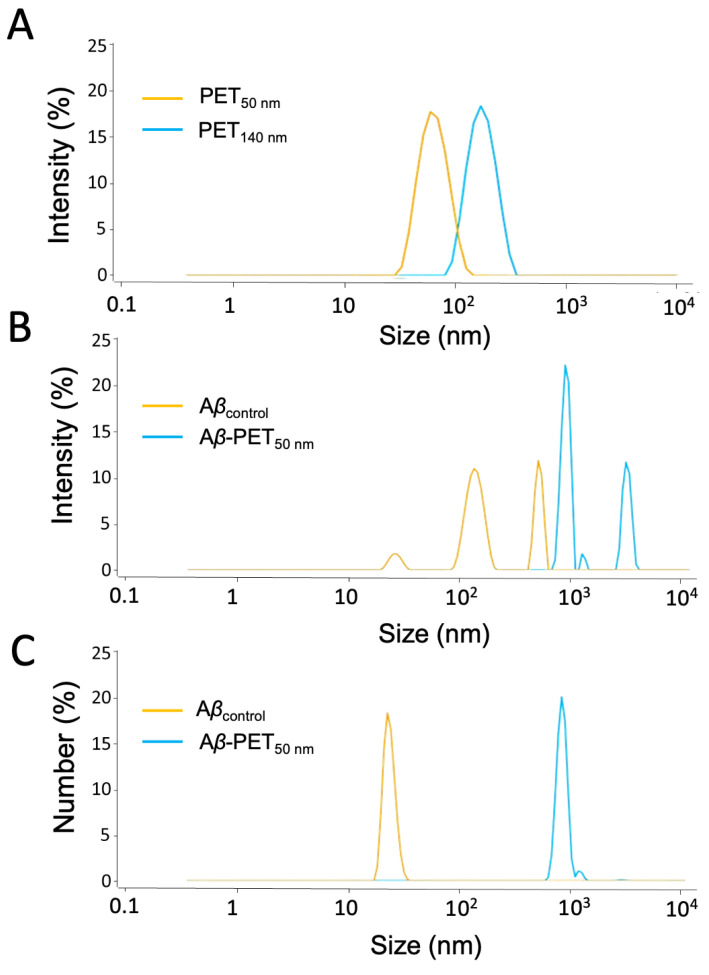
Particle size of PET NPs and Aβ-PET NPs determined using DLS and MADLS. (**A**) PET NPs of two different particle size; (**B**) intensity-weighted size distribution of Aβ (orange) and Aβ-PET NPs (blue); (**C**) number-weighted size distribution of Aβ (orange) and Aβ-PET NPs (blue).

The size of PET nanoparticles (NPs) in both the absence and presence of amyloid-beta (Aβ) peptides was measured using multi-angle dynamic light scattering (MADLS). Size distributions of PET NPs (PET_50nm_ and PET_140nm_) are shown in Figure 1. The Aβ solution (Aβ_control_) showed particles of around 22 nm, 130 nm, and 430 nm, indicating different stages of Aβ aggregation, from smaller oligomers to larger structures. When Aβ was incubated with PET_50nm_, additional peaks around 140 nm and 1000 nm appeared, suggesting that PET NPs promote further aggregation of Aβ. Number-weighted analysis indicated that the Aβ_control_ sample mostly contained small particles, around 22 nm in size. The addition of PET_50nm_ resulted in a shift towards larger particles, with peaks at 140 nm and 1000 nm (Figure 1C). Incubation of Aβ with PET_140nm_ also resulted in a shift towards larger particles (Figure S1).

PET NPs exhibit negative zeta potentials, with PET_50nm_ showing a zeta potential of −41 ± 2 mV and PET_140nm_ showing a zeta potential of −15 ± 1 mV (Table S1). Smaller particles tend to have a higher surface charge density, which results in more negative zeta potential [26]. This effect is due to the increased surface area-to-volume ratio in smaller particles. The zeta potential values indicate changes in the stability of the NPs upon interaction with Aβ. The zeta potential of the Aβ_control_ was −30 ± 2 mV. When Aβ was combined with PET NP to form Aβ-PET_50nm_, the zeta potential increased to −20 ± 2 mV (Table S1) suggesting a decrease in stability over time due to presence of these PET NPs and further leading to aggregation and larger particle sizes. The electrostatic repulsion between PET_50nm_ particles itself may also be weakened in the presence of an Aβ protein environment, leading to the clumping of nanoparticles, as the attractive forces become stronger than the repulsive forces.

Although the exact binding mechanisms between peptides and PET NPs are not fully understood, recent studies using other nanoparticles indicate that these interactions are primarily mediated by weak forces such as hydrophobic interactions, hydrogen bonds, Van der Waals attraction forces, and electrostatic forces [27]. It has been shown that when NPs enter a biological environment, peptides and other biomolecules adsorb to their surfaces, forming a “peptide corona” [28,29]. This layer significantly alters the physicochemical properties of the NPs and their interactions with other proteins [30,31]. The surfaces of the NPs may undergo alterations in response to the adsorption of peptide molecules, which could influence the overall reactivity of the NPs [31,32]. The formation of the peptide corona is influenced by several factors, including size, shape, and chemical composition of the NPs, the type of medium (including the types of peptides and other chemical species present), the duration of exposure, and the ratio of NPs to peptides [33,34,35].

### 2.2. Aβ Fibrillation Kinetics

Amyloid-beta (Aβ) is an amphipathic peptide that tends to self-aggregate, forming structures such as oligomers and fibrils [36]. Typically, Aβ aggregation is characterized by an increase in β-sheet structure and the formation of the cross-β structure [37]. The fibril formation process starts with a slow interaction between misfolded peptides and preformed oligomers, which act as nuclei for fibril elongation [38]. The kinetics of peptide fibrillation include three phases: the lag (nucleation) phase, the elongation phase, and the saturation phase, often displaying a sigmoidal growth curve [39]. The nucleation phase is the activation time required for seed formation, from which fibrillation begins. The thioflavin T (ThT) fluorescence assay is used to monitor the impact of PET NPs on amyloid fibril formation. The lag phase, characterized by an initial slow increase in ThT fluorescence intensity, is indicative of the nucleation process before rapid fibril growth occurs.

**Figure 2:**
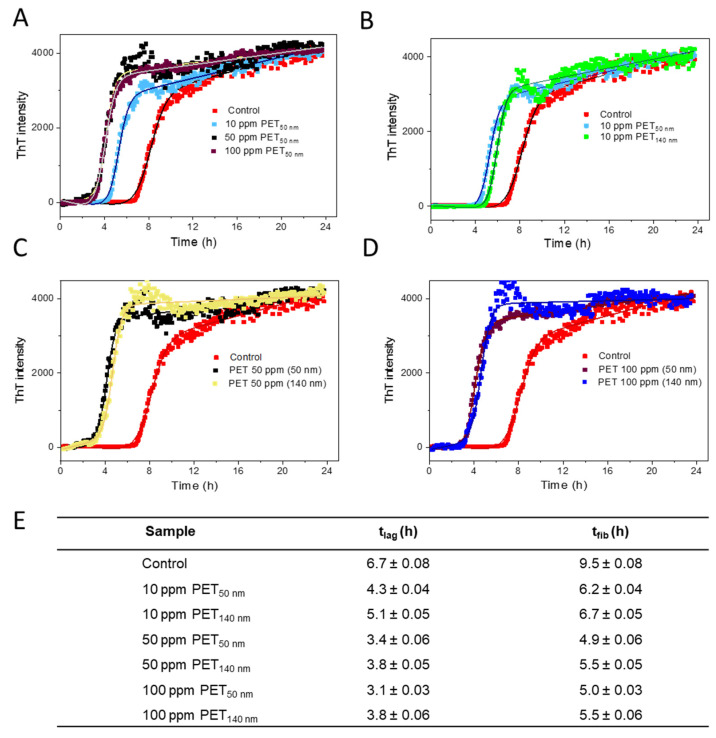
Fibrillation kinetics of Aβ_control_ and Aβ-PET NPs (PET_50nm_ and PET_140nm_) measured by ThT fluorescence. (**A**) Concentration- and size-dependent (**B**–**D**) effect of PET NPs on kinetics of Aβ. (**E**) The concentration- and size-dependent characteristic lag time (t_lag_) and fibrillation time (t_fib_). The peptide concentration was 0.125 mg/mL (28 µM) at pH 7.4 at a temperature of 37 °C. Experimental data were fitted (as described in the Section 3 and Supplementary Figure S2) to sigmoidal functions represented by the equation: *I =* $y_{i}+\frac{y_{f}}{1+e^{-}\frac{\left(t-t_{0}\right)}{\tau }}$.

The time-dependent ThT fluorescence intensities of Aβ_control_ and Aβ treated with either PET_50nm_ or PET_140nm_ at various concentrations are presented in Figure 2. The presence of both PET_50nm_ and PET_140nm_ decreases the lag phase time as well as fibrillation time for Aβ, with clear trends observed across different concentrations. Aβ_control_ shows a lag time of 6.7 ± 0.1 h, while the lag time is slightly reduced for 10 ppm PET_50nm_ (4.3 ± 0.1 h) and significantly reduced for 100 ppm PET_50nm_ (3.1 ± 0.1 h) (Figure 2A). Similarly, the effect of concentration of PET on fibrillation time has been observed. As shown in Figure 2E, the fibrillation time is short for 50 ppm and 100 ppm PET_50nm_ (5.0 ± 0.1 h), slightly longer for 10 ppm PET_50nm_ (6.2 ± 0.1 h) and longest for Aβ_control_ (9.5 ± 0.1 h) (Figure 2E). Interestingly, our observations showed that both the lag time and the fibrillation time are slightly shorter for PET_50nm_ (in all concentration groups) compared to larger PET_140nm_ (Figure 2B–D). The fibrillation time is increased as the concentration of PET NPs increased. However, at higher concentrations, the larger particles exhibited a saturation effect, where additional increases in concentration (e.g., 50 and 100 ppm) did not further enhance the fibrillation kinetics. Zeta potential measurement clearly showed that our PET NPs are negatively charged. It is possible that the acceleration of the Aβ aggregation process by PET NPs is due to electrostatic interactions of negatively charged PET NPs with positively charged residues in the Aβ sequence (e.g., Arg_5_, His_6_, His_13_, His_14_, Lys_16_, Lys_28_).

Our results showed that the particle size of PET NPs have an impact on the kinetics of Aβ aggregation. It is conceivable that NPs act as efficient nucleation seeds, thereby accelerating the process of fibril growth. Thus, NPs can shorten the lag phase by providing catalytic surfaces that facilitate nucleation [40,41]. Previous studies have shown that polystyrene nanoparticles (PS NPs) accelerate the nucleation rate, increase the production of oligomers, and enhance the content of antiparallel β-sheets of Aβ_40_ peptide [21]. Likewise, it has been demonstrated that polyethylene nanoparticles (PE NPs) interact with secondary structure elements, promoting the formation of β-sheets [42]. Another kinetics study with amino-modified PS NPs showed that the fibrillation process of Aβ_40_ peptides is accelerated at NPs concentrations of 20 ppm and 50 ppm, resulting in a reduced lag phase [43]. Our results are consistent with these findings, suggesting the role of PET NPs in promoting β-sheet formation and accelerating fibril growth. However, unlike previous studies, where polystyrene did not influence the nucleation rate when higher concentrations of Aβ were used (>20 µM) [21], we see that PET NPs significantly influences the nucleation rate even at a high concentration of Aβ (28 µM), as used in our study. Interestingly, while other investigations have seen that PS NPs promote more oligomers in Aβ solution during aggregation but inhibit higher-order fibrils formation [21], we observed that PET NPs also promote both aggregation and higher-order fibril formation.

### 2.3. Secondary Structure Analysis

**Figure 3:**
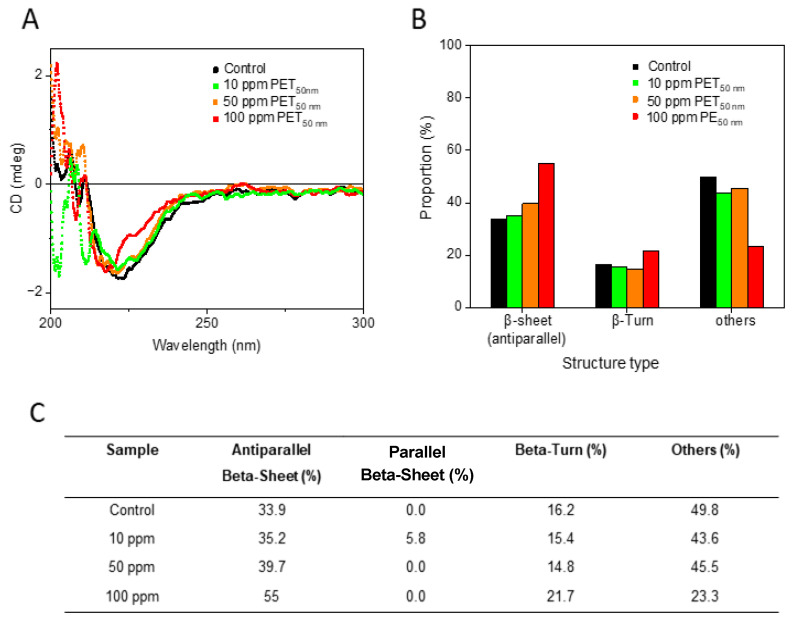
Circular dichroism (CD) spectroscopy analysis of Aβ and Aβ with concentrations 0, 10, 50, and 100 ppm of PET50 nm. (**A**) CD spectra of Aβcontrol and Aβ-PET_50nm_ 10 ppm, 50 ppm, and 100 ppm. (**B**) Quantitative analysis of the secondary structure content from CD data analyzed using BeStSeL. The bar graph shows the percentage of antiparallel β-sheet, β-turn, and other structures in Aβ and Aβ-PET_50nm_. (**C**) Table derived from quantitative analysis results analyzed using BeStSeL (see Supplementary Figure S3).

To gain insight into the conformation of Aβ aggregates, circular dichroism (CD) spectroscopy studies were performed (Figure 3). The CD spectra of Aβ_control_ and of varying concentrations of Aβ-PET_50nm_ (0, 10, 50, and 100 ppm) are shown in Figure 3A. The negative peak around 220 nm is indicative of β-sheet structure. As the concentration of PET_50nm_ increases, there is a notable change in the negative peaks and the shape of the curves. The corresponding quantitative analysis of the secondary structure content, derived from the CD spectra, is shown in Figure 3B. As the concentration of PET_50nm_ increases, there is a significant rise in antiparallel β-sheet content, from 33.9% in the Aβ_control_ to 55% in the presence of 100 ppm PET_50nm_. (Figure 3C). This signifies a conformational shift that may be driven by the interaction between PET_50nm_ and specific regions of the Aβ peptide, disrupting its natural folding pathways [44]. This indicates an increase in structural disorder, which suggests that while some parts of the peptide become more ordered, others become more flexible. This flexibility could make certain regions of the peptide more accessible for interaction with other molecules and influence the aggregation behavior of the peptide. An increase in β-turn structures from 16% to 22% and a significant reduction in other structures from 49.8% to 23.3% further highlight the conformational specificity induced by PET_50nm_ (Figure 3C). The loss of these diverse structural elements indicates that PET_50nm_ may drive Aβ peptides towards an aggregation-prone conformation.

Our results are consistent with those of a previous study on another type of nanoplastic, PS NPs, which demonstrated a twofold increase in antiparallel β-sheet content in Aβ aggregates [21]. In addition, the change in β-turns observed with PS NPs in that study, similar to the structural changes we observed with PET_50nm_, suggests that these nanoparticles induce regions of the peptide to become more flexible.

The CD data showed that PET_50nm_ at higher concentrations altered the secondary structure of Aβ peptides by promoting the formation of antiparallel β-sheets and reducing the content of β-turns and other structures. These structural changes enhance the nucleation and aggregation processes, aligning with our observation of the accelerated fibrillation kinetics at the elevated PET_50nm_ concentrations (50 and 100 ppm).

**Figure 4:**
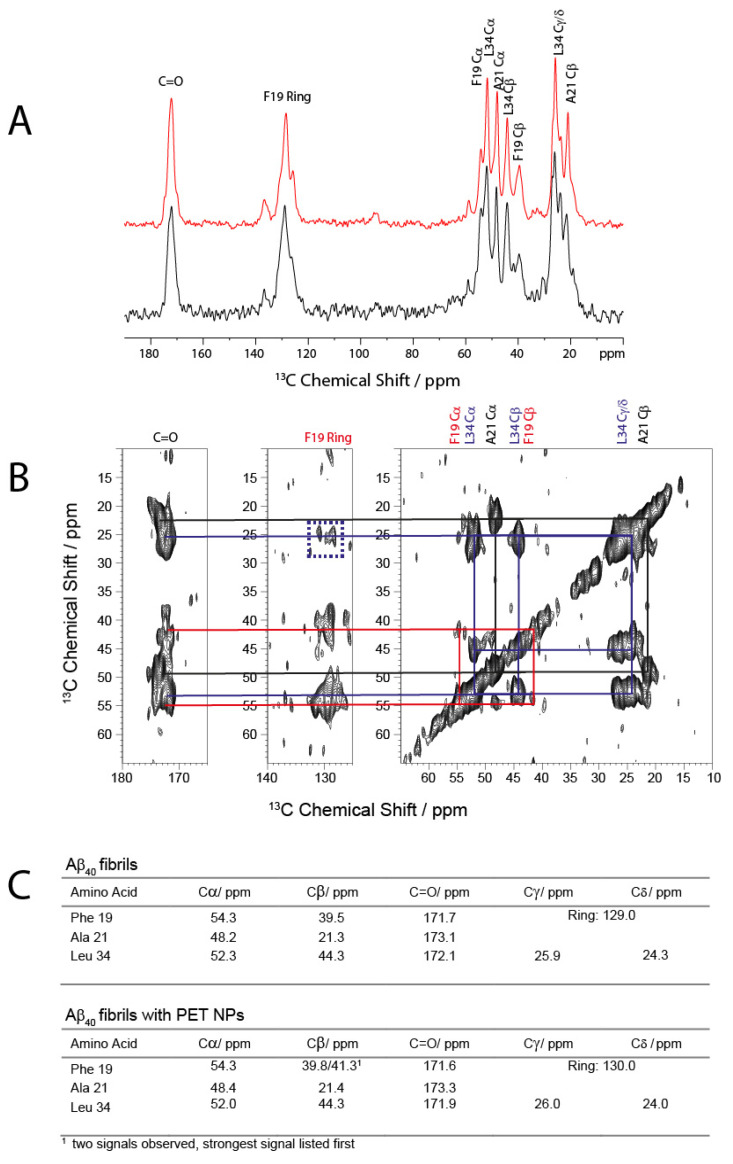
(**A**) 1D ^13^C CP MAS NMR spectra of the Aβ_40_ in the absence (red) and presence of 100 ppm PET_50nm_ (black), (**B**) ^13^C-^3^C 2D DAAR NMR spectrum of the Aβ_40_ fibrils in the presence of 100 ppm PET_50nm_. The intraresidual cross peaks are labeled for Phe_19_ (red), Ala_21_ (black), and Leu_34_ (blue). The blue dotted box indicates the inter-residual cross peak between the side chain of Leu_34_ and the aromatic ring of Phe_19_. All spectra were measured at 30 °C and a MAS frequency of ~11.7 kHz. (**C**) Table showing isotropic chemical shifts (relative to TMS) for Aβ_40_ fibrils in the absence or presence of 100 ppm PET_50nm_.

To further investigate if the effect of PET NPs on fibrillation kinetics causes changes in the local structures within the cross-β structure of amyloid fibrils, we performed solid-state NMR measurements. To this end, we used Aβ_40_ peptides isotopically labeled with ^13^C/^15^N for Phe_19_, Ala_21,_ and Leu_34_ to gain insights into some key features of the Aβ_40_ structure in fibrils. The 1D ^13^C CP MAS NMR spectrum of the fibrils grown in the presence of the PET_50nm_ NPs is very similar to the spectrum of the fibrils in absence of the NPs (Figure 4A). From the 2D DARR NMR spectrum (Figure 4B), the isotropic ^13^C chemical shifts for the labeled amino acids were obtained, which are sensitive to the secondary structure [45]. These values (Figure 4C) correspond very well with the chemical shifts observed in other studies on Aβ_1–40_ and the known β-sheet structures typical for Aβ in the respective regions [46,47,48,49]. In addition, the well-known inter-residual contact between Phe _19_ and Leu_34_ is observed through cross peaks between the side chain of Leu_34_ and the aromatic ring of Phe _19_ (Figure 4B) [46,47,48,49,50,51]. Overall, the ^13^C MAS NMR results show that the presence of PET NPs during fibrillation results in fibrils which exhibit a structure similar to the known Aβ_1–40_ fibrils, thus indicating that the local structure within a cross-β structure of amyloid fibrils was not affected by the presence of PET_50nm_ NPs during fibrillation.

### 2.4. Morphology of Aβ Fibrils 

**Figure 5:**
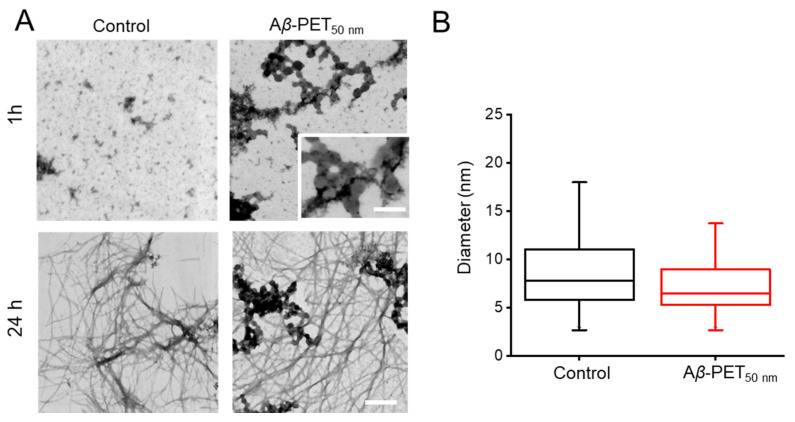
Negatively stained images observed using TEM. (**A**) TEM images of Aβ and Aβ-PET_50nm_ NPs at two different time points (1 h and 24 h). (**B**) Box plots of measured fibril diameters (n = 100, a total of 100 measurements from two independent preparations). Scale bars: 250 nm and 100 nm (inset).

The morphology of Aβ fibrils in the presence and absence of PET NPs was investigated using Transmission Electron Microscopy (TEM) (Figure 5). The effect of PET_50nm_ on aggregation and fibrillation initially evaluated at two different time points are shown in Figure 5A. At 1 h, Aβ_control_ contains small spherical aggregates of varying size, some of which cluster together. Interestingly, the introduction of 100 ppm PET_50nm_ NPs led to the formation of dense aggregates surrounding the nanoparticles. This might be due to interaction with the Aβ fibrils, as highlighted by the inset (inset Figure 5A). Moreover, when considering the MADLS data (Figure 1) in concert with these findings, one can infer that a high fraction of peptide is attached to the surface of the PET NPs that contribute to the acceleration of early aggregation process of Aβ. By 24 h, in both Aβ_control_ and Aβ-PET_50nm_, fibrils are formed. Furthermore, the fibril diameters were quantitatively assessed from TEM images (Figure 5B). The box plot comparison shows no significant difference in the average fibril diameter between Aβ alone and Aβ incubated with PET NPs (*p* > 0.05). The fibril diameter of 100 ppm Aβ-PET_50nm_ is slightly smaller than Aβ_control_. The findings revealed that while PET NPs influenced the lag time and fibrillation time, it did not stop the formation of fibrils in both the Aβ_control_ and the 100 ppm Aβ-PET_50nm_ samples. Furthermore, it did not result in a notable change in the diameter of the fibrils.

## 3. Materials and Methods

### 3.1. Chemicals

All chemicals were purchased from Sigma-Aldrich (St. Louis, MO, USA) unless otherwise stated.

### 3.2. Preparation of PET NPs

Different sizes of PET NPs were prepared based on the previously described method [24,52]. Briefly, 100 mg of amorphous PET (Goodfellow, Germany) was dissolved in 10 mL of hexafluoroisopropanol (HFIP) (1% *v*/*v*) at room temperature over a period of 24 h. The resulting PET suspension was transferred to a buret and added dropwise into 200 mL of ice-cooled deionized (DI) water and stirred at different speeds to prepare different sizes of NPs (100 rpm for 140 nm NPs and 600 rpm for 50 nm NPs). Then, larger particles were removed from the suspension using a cellulose nitrate membrane filter with a pore size of 0.2 µm (Sartorius, Göttingen, Germany). The HFIP was then removed from the solution using a rotary evaporator (Heidolph Instruments, Wood Dale, IL, USA) at 50 °C and reduced pressure (~250 mbar). The NPs were allowed to settle in a cylinder for 2 h, after which the suspension was collected. The concentration of NPs was determined gravimetrically by drying 2 mL of the suspension on a pre-dried polymer pellet (triplicates) at 50 °C for 24 h and then weighing the residue. The prepared nanoparticle solution was stored at room temperature, and it was stable for several weeks without any precipitation.

### 3.3. Peptide Synthesis

Peptide synthesis was performed by the Peptide Synthesis Core Unit of Leipzig University (https://home.uni-leipzig.de/izkf/indexpeptide.html, accessed on 7 January 2025). Standard Fmoc solid-phase synthesis was used to produce the Aβ_40_ peptides with the WT sequence DAEFRHDSGY EVHHQKLVFF AEDVGSNKGA IIGLMVGGVV. The peptide purity level was determined via HPLC analysis and MALDI mass spectrometry was ≥97% depending on the peptide (see Supplementary Information Figure S4).

### 3.4. Aβ_40_ Fibril Preparation

The peptide powder was dissolved in dimethyl sulfoxide (DMSO) (approximately 0.5 mg of peptide in 20 μL DMSO), incubated at room temperature for about 30 min, and then diluted to a concentration of 1 mg/mL using an aqueous buffer. The buffer consisted of 25 mM sodium phosphate, 150 mM NaCl, at pH 7.4. Afterwards, the peptide solution was further diluted to 0.125 mg/mL with aqueous buffer. To this mixture, different sizes (50 and 140 nm) and concentrations (0, 10, 50, and 100 ppm) of PET_50nm_ and 20 µM ThT were added. A control experiment was performed by incubating Aβ monomers without PET_50nm_, providing baseline data to compare the impact of PET NPs on Aβ aggregation. Six aliquots (125 μL) of final solution were transferred into a 96-well plate (Corning^®^ 96-well half-area microplate, polystyrene with nonbinding surface coating, black, flat bottom clear). The dead time between the preparation of the measuring solution and the first measurement was less than 5 min. ThT fluorescence was recorded using a microplate reader (Tecan Infinite M200, Tecan Group AG, Mannedorf, Switzerland) at 37 °C, with an excitation wavelength of 440 nm and an emission detection wavelength of 482 nm. The measurements included a shaking/5 min waiting cycle and were taken every 5 min for 24 h.

### 3.5. Dynamic Light Scattering (DLS)

Hydrodynamic diameters and zeta potentials of Aβ_40_ in the presence and in the absence of PET NPs were measured using a Zetasizer Nano ZS, Malvern, UK. Measurements were made by diluting the peptide (0.125 mg/mL) with and without PET NPs (50 nm, 100 ppm) in the water (DI, 1:2 ratio) at pH 7.4 at 25 °C. The laser light was set at 633 nm. Multi-Angle Dynamic Light Scattering (MADLS) measurements were performed to collect the intensity of backscattering, forward scattering, and side scattering. Each measurement consisted of 100 scans. Zeta potential measurements were recorded for PET NPs (50 nm, 140 nm) as well as Aβ with and without PET_50nm_**.** Three independent measurements were performed, and the zeta potential was calculated based on the electrophoretic mobility of sample particles. Refractive indices were 1.330 for water and 1.45 for the protein, respectively, for the polydispersant and material. Data was processed with the Malvern Zetasizer Software (version 3.30) (Malvern Instruments, Worcestershire, UK).

### 3.6. Transmission Electron Microscopy

A volume of 2 μL of a diluted fibril solution from the final state of ThT measurements (1:20 (*v*/*v*) with ddH_2_O) was transferred onto a formvar film-coated copper grid. After evaporating the solvent, the sample was stained with 1% uranyl acetate. Images were captured using a Zeiss SIGMA electron microscope equipped with a STEM detector and operated with Atlas software 5 (Zeiss NTS, Oberkochen, Germany). The diameters of amyloid fibrils seen on the TEM images were measured using ImageJ (version 1.53e; National Institute of Health, Bethesda, MD, USA) software. Horizontal lines of equal distance were drawn on the image, and the diameter of the fibrils intersecting the lines was measured. A total of 100 random different locations along the fibrils for each group of samples were analyzed. The mean ± one standard deviation (SD) was then calculated. Statistical significance was assessed using a one-way ANOVA test (*p* > 0.05 considered as the significance difference between groups), and a box plot was constructed from these data using OriginPro v.8 (OriginLab, Northampton, MA, USA).

### 3.7. Circular Dichroism (CD) Spectroscopy

For stock solution preparation, approximately 1 mg lyophilized peptide powder was presolubilized in 40 μL DMSO and diluted to a concentration of 1 mg/mL using standard phosphate buffer (25 mM sodium phosphate, 150 mM sodium chloride, pH 7.4) with and without PET_50nm_ (10, 50, and 100 ppm). For fibril measurements, the stock solution was incubated for 24 h at 37 °C and 450 rpm using a thermoshaker prior to dilution to measure concentration. Before dilution to the measured concentration, the fibrils were ultra-sonicated in a water bath for 30 min and then vigorously vortexed. For recording CD spectra, the stock solution was diluted to a final peptide concentration of 10 μg/mL with DI water. CD spectra were recorded on a Jasco J-1500 spectrophotometer in a 1 mm cuvette at 25 °C. The data was acquired at 50 nm/min with a data pitch of 0.1 nm, a bandwidth of 2 nm, and 4 s data acquisition time. The phosphate buffer solution was measured separately and was automatically subtracted as a baseline. The proportion of each secondary structure component was further analyzed using the Beta Structure Selection (BeStSel). This method offers enhanced β-structure determination by considering the parallel or antiparallel orientation and the twist of β-sheets, resulting in more accurate performance for all secondary structure types [53].

### 3.8. Solid-State NMR-Spectroscopy

For solid-state NMR measurements, the fibrils were prepared as before for the CD measurements using an Aβ_40_ peptide isotopically labeled with ^13^C/^15^N for the amino acids Phe_19_, Ala_21_, and Leu_34_. At the end of the fibrillation, the sample was ultracentrifuged (200,000× *g* for 2 h). The pellet was lyophilized overnight, rehydrated with 50 wt% H_2_O, homogenized by ten freeze-thaw cycles, and finally transferred into 3.2 mm MAS NMR rotors. Solid-state MAS NMR spectra were acquired on a Bruker 600 Avance III NMR spectrometer (Bruker BioSpin GmbH, Rheinstetten, Germany) at a resonance frequency of 600.3 MHz for ^1^H and 150.96 MHz for ^13^C, using a triple-channel 3.2 mm MAS probe at a temperature of 30 °C and a MAS frequency of 11.7 kHz. The 90° pulse lengths were 4 µs for ^1^H and for ^13^C. The ^13^C MAS chemical shifts were referenced externally relative to TMS. ^13^C-^13^C DARR NMR spectra were acquired with a mixing time of 500 ms to observe inter-residual interactions.

### 3.9. Fluorescence Intensity Data Evaluation

Fluorescence intensity data were normalized by the equation *I =* $\frac{{(I}_{t}-I_{min)}}{I_{max}}$, where *I* represents the normalized intensity, *I_t_* is the measured intensity value at the corresponding time point, and *I_min_* and *I_max_* are the minimal and maximal intensities measured for the corresponding well, respectively. Normalized data were fitted using a simple sigmoidal curve model, *I =*${\;y}_{i}+\frac{y_{f}}{1+e^{-}\frac{\left(t-t_{0}\right)}{\tau }}$, where *I* is the normalized intensity, *t* is the time, *t_0_* is the time at half-maximal intensity, and *τ* is a measure of the fibrillation time [54]. The lag time was calculated as t*_lag_* = *t*_0_ − 2*τ*, and the fibrillation time as t*_fib_* = *4τ.* Mean values and standard deviations of lag time and fibrillation time were calculated. The statistical analysis was carried out using OriginPro v.8 (OriginLab, Northampton, MA, USA). A significance level of *p* < 0.05 was used to determine statistical significance.

## 4. Conclusions

In conclusion, our findings show that PET NPs significantly accelerate the aggregation process of Aβ peptides, as evidenced by a reduction in both the lag time and the fibrillation time, particularly at a concentration of 100 ppm PET NPs (both PET_50nm_ and PET_140nm_). Moreover, our secondary structure analysis using CD spectroscopy demonstrated that PET_50nm_ induces a conformational shift in Aβ peptides, favoring the formation of antiparallel β-sheets while increasing structural heterogeneity. This shift is accompanied by a reduction in other secondary structures, suggesting that PET NPs promote a more uniform and aggregation-prone conformation. Despite these alterations, TEM analysis indicated that while PET_50nm_ modulates the aggregation kinetics, they do not significantly affect the final fibril morphology or diameter. ^13^C solid-state NMR spectroscopy revealed that the local structure of the cross-β configuration in amyloid fibrils was not affected by the presence of PET_50nm_ nanoparticles during fibrillation. Taken together, these results show that PET NPs influence the aggregation behavior of Aβ peptides and may potentially exacerbate neurotoxic effects associated with AD. These findings raise concerns about the potential environmental and health impacts of PET NPs, emphasizing the need for further investigation into their long-term effects on neurodegenerative diseases.

## Data Availability

All relevant data are within the paper and its Supporting Information Files.

## Supplementary Materials

The following supporting information can be downloaded at https://www.mdpi.com/article/10.3390/molecules30071432/s1: Figure S1: Particle size of Aβ-PET NP_140nm_ determined by DLS and MADLS; Figure S2: A representative fluorescence time evolution fitting and explanation of Aβ_control_; Figure S3: The proportion of each secondary structure component analyzed from CD spectrum using the BeStSel; Figure S4: Mass spectrum ESI-MS of Amyloid beta 1-40. Table S1: Zeta potential values for PET NPs (50 nm, 140 nm) and Aβ with and without PET NPs.
