# Supplementary material for: The Effect of Polyethylene Terephthalate Nanoplastics on Amyloid-β Peptide Fibrillation
Source: Molecules. 2025 Mar 24;30(7):1432. doi: 10.3390/molecules30071432 (PMC11990616; doi:10.3390/molecules30071432)
Supplement: Supplementary file 1 [file molecules-30-01432-s001.zip › molecules-3523531-supplementary.pdf]

molecules

MDPI

Supporting information

Table S1. Zeta potential values for PET NPs (50 nm, 140 nm) and Aβ with and without PET NPs, measured using a Zetasizer Nano ZS. Values represent Mean ± SD (n=3).

Sample	Zeta potential (mV)
$PET_{50\ nm}$	-41 ± 1.6
$PET_{140\ nm}$	-15 ± 1.4
Aβ control	-30 ± 2.0
Aβ-$PET_{50\ nm}$	-20 ± 1.7

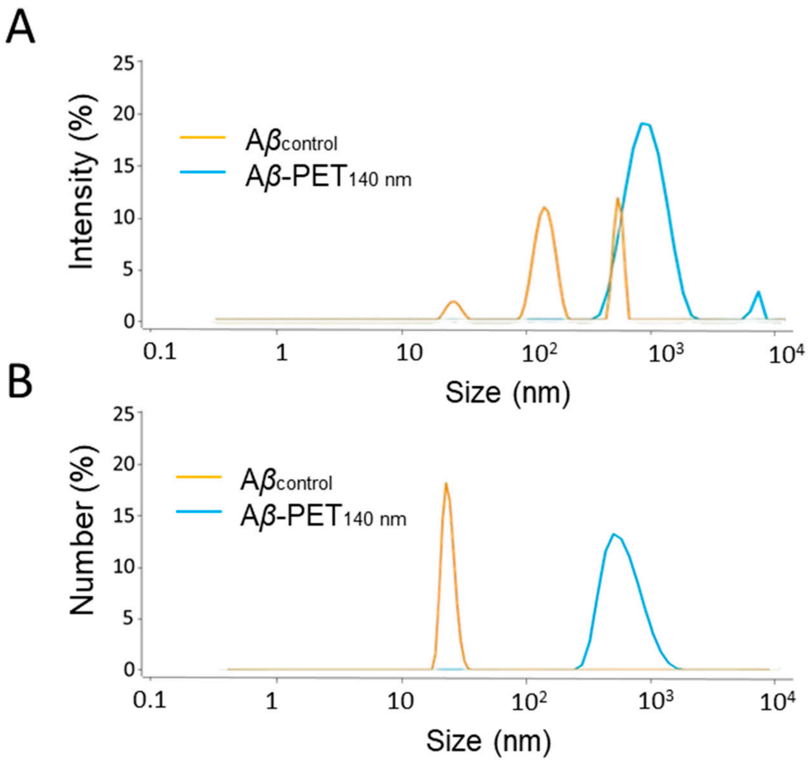

Figure S1. Particle size of Aβ-PET NP_{140nm} determined by DLS and MADLS. (A) Intensity weighted size distribution of Aβ (orange) and Aβ-PET NP_{140nm} (blue). (C) Number weighted size distribution of Aβ (orange) and Aβ-PET NP_{140nm} (blue).

A

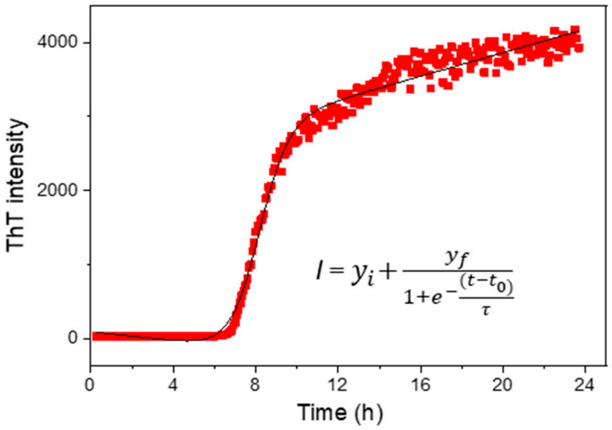

B

Equation	$I = y_i + \frac{y_f}{1+e^{-\frac{(t-t_0)}{\tau}}}$						
Plot	Control	10 ppm $PET_{50\ nm}$	10 ppm $PET_{140\ nm}$	50 ppm $PET_{50\ nm}$	50 ppm $PET_{140\ nm}$	100 ppm $PET_{50\ nm}$	100 ppm $PET_{140\ nm}$
I_{min}	91,95601	93,54637	72,96833	72,50379	59,14945	205,91147	103,56504
I_{max}	2179,1846	2031,3463	2404,86414	2252,94558	2694,54052	2662,40054	2352,91868
m_1	-31,33665	-32,74922	-15,18814	37,32636	50,87939	-68,13628	33,05018
m_2	111,06181	92,93238	67,52245	-12,75113	-41,76584	102,61822	-28,49383
t_0	8,11028	5,26331	5,94252	4,15954	4,68587	4,0372	4,66453
τ	0,71357	0,46079	0,41378	0,39551	0,41086	0,48	0,44141

C

Parameter	Notation	Description
Minimum fluorescence intensity before aggregation starts	I_{min}	Represents the baseline fluorescence intensity prior to the onset of aggregation.
Maximum fluorescence intensity at fibril saturation	I_{max}	Indicates the peak fluorescence intensity when fibril formation reaches completion.
Slope of the initial phase	m_1	Reflects the rate of aggregation during the early stage of fibril formation.
Slope of the elongation phase	m_2	Represents the rate of aggregation during the fibril growth phase.
Time at half-maximal intensity	t_0	Marks the inflection point of the sigmoidal curve, indicating when half of the maximum fluorescence is achieved.
Fibrillation time constant	τ	Denotes the time scale over which the aggregation process occurs.
y_i	$y_i=I_{min}+m_1$	Corresponds to the baseline intensity
y_f	$y_f=I_{max}+m_2$	Corresponds to the fluorescence increase
Time before significant fibril formation begins	$t_{lag}=t_0-2\tau$	Represents the lag phase duration before noticeable fibril formation.
Total time for fibril formation	$t_{fib}=4\tau$	Indicates the overall duration required for complete fibril formation.

Figure S2. (A) A representative fluorescence time evolution fitting of $A\beta_{control}$; **(B)** The table showing the analysis of the kinetic parameters describing the aggregation of fibrils under different concentrations (10 ppm, 50 ppm, and 100 ppm) and sizes (50 nm and 140 nm) of PET nanoplastics. The parameters are derived from fluorescence intensity measurements and fitted to a sigmoidal model, represented by the equation: $I = y_i + \frac{y_f}{1+e^{-\frac{(t-t_0)}{\tau}}}$; **(C)** Explanation of parameters from kinetic measurements.

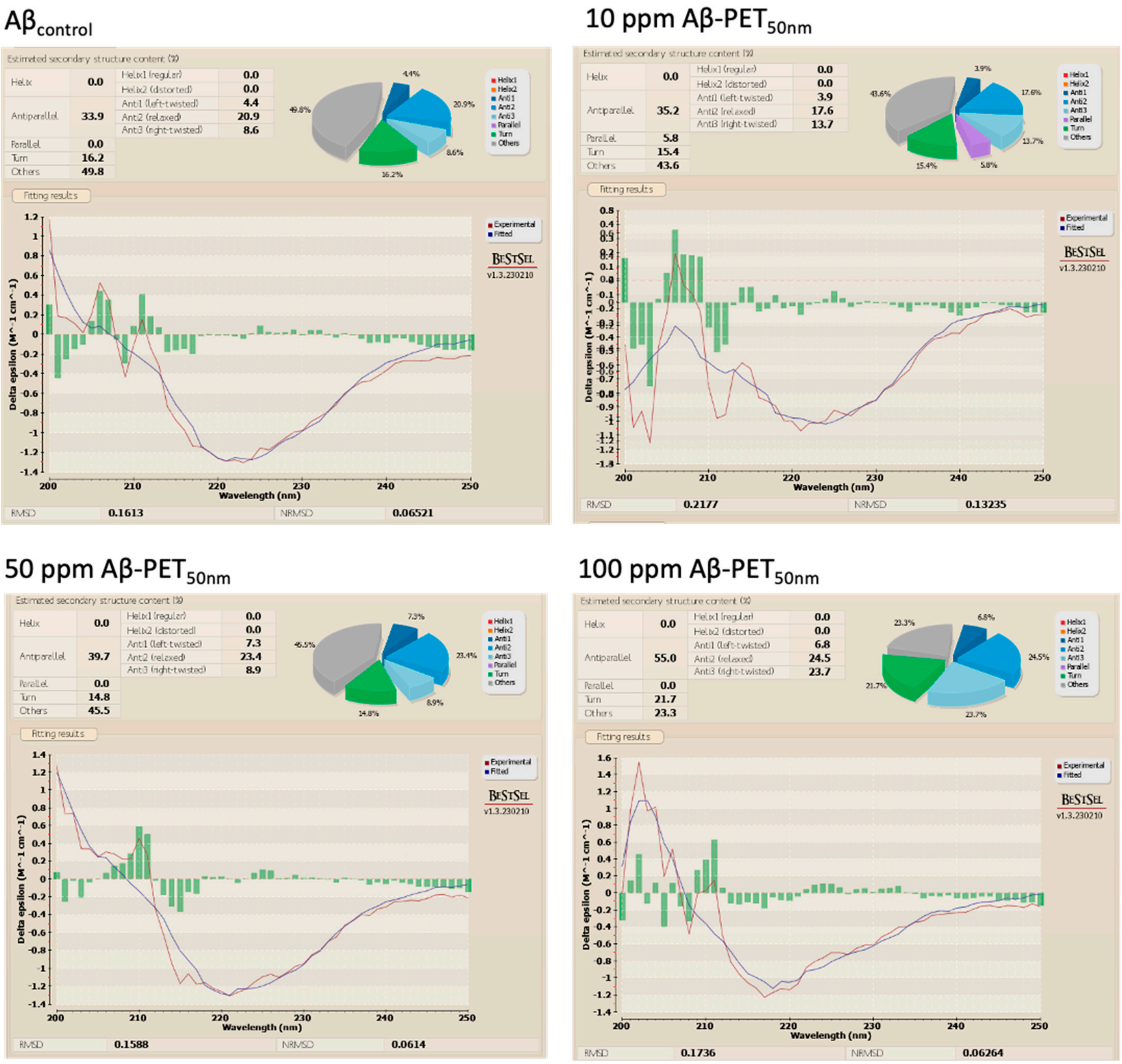

Figure S3. The proportion of each secondary structure component analyzed from CD spectrum using the BeStSel. The NRMSD values of $A\beta_{control}$ and 10, 50 and 100 ppm of $A\beta\text{-}PET_{50nm}$ are 0.06521, 0.13235, 0.0614 and 0.06264, respectively.

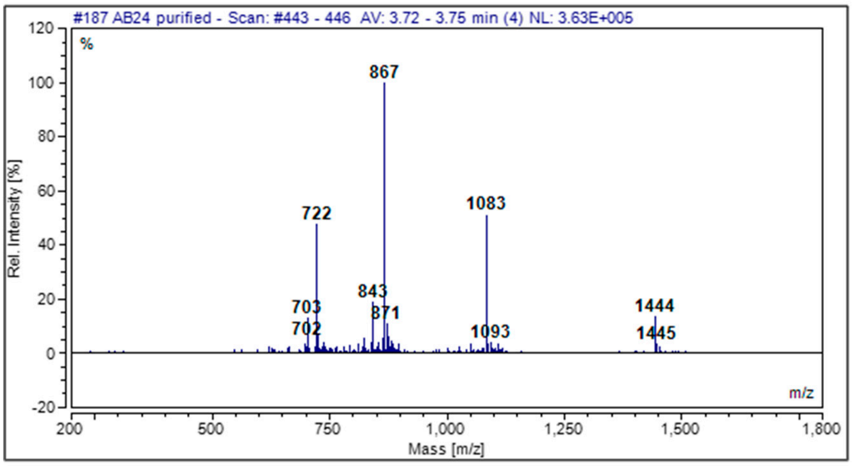

MW:	4329	M+1H
Ladung z	m/z	
1	4330	M+1H
2	2166	M+2H
3	1444	M+3H
4	1083	M+4H
5	867	M+5H
6	723	M+6H

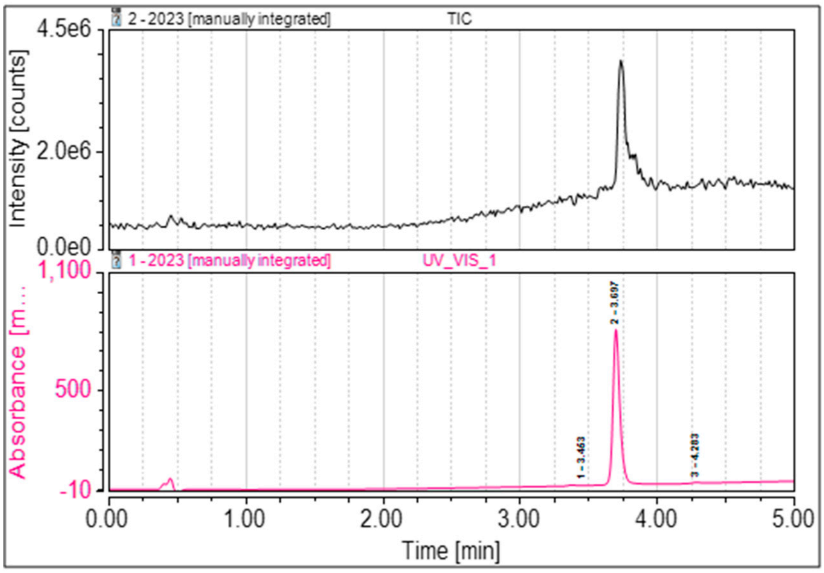

No.	Ret.Time	Peak Name	Height	Rel.Area	Area	Amount	Type
	min		mAU	%	mAU*min	n.a.	
1	3,45		2,2	0,34	0,16	n.a.	BMB*
2	3,70		784,5	98,77	45,12	n.a.	BMB*
3	4,28		5,2	0,88	0,40	n.a.	BMB*
Total:			791,835	100,000	45,68	0,000	

Figure S4. Mass spectrum ESI-MS of Amyloid beta 1-40. The asterisk (*) refers to peaks that were manually integrated using the Baseline-Manual Baseline (BMB) method in the chromatographic analysis.
